# Kaempferol as a multifaceted immunomodulator: implications for inflammation, autoimmunity, and cancer

**DOI:** 10.3389/fimmu.2025.1671519

**Published:** 2025-08-21

**Authors:** Han Dong, Ge Song, Zhe Wang, Xue Wu, Qi Wang, Yue-Hui Wang

**Affiliations:** ^1^ Department of Geriatrics, Jilin Geriatrics Clinical Research Center, The First Hospital of Jilin University, Changchun, Jilin, China; ^2^ Department of Internal Medicine, Zaduo County People’s Hospital, Yushu, Qinghai, China; ^3^ Department of Gerontology, The Second Medical Center & National Clinical Research Center of Geriatric Diseases, Chinese People’s Liberation Army (PLA) General Hospital, Beijing, China

**Keywords:** Kaempferol, modulation, T cells, natural killer cells, dendrite cells, macrophages, granulocytes

## Abstract

Kaempferol (KMF) is a dietary flavonoid exhibiting profound immunomodulatory effects across multiple immune cell populations. This review synthesizes current insights into how KMF regulates diverse immune cell populations and its therapeutic potential in inflammatory and immune-related disorders. KMF exhibits multifaceted effects on T cells. It inhibits T cell activation via suppressing various signaling pathways and calcineurin. Additionally, it regulates T cell subset balance through the modulation of different transcription factors. In natural killer (NK) cells, KMF enhances proliferation and cytotoxicity. This effect is partly mediated by gut microbiota modulation, which further boosts anti-tumor immunity. For dendritic cells (DCs), KMF shows context-dependent effects. It can promote adaptive immunity in some settings, while in inflammatory contexts, it suppresses DC maturation and cytokine secretion. KMF reduces neutrophil infiltration and the formation of neutrophil extracellular traps (NETs). It also alleviates eosinophil-driven allergic inflammation and blocks mast cell degranulation. Regarding macrophages, KMF shifts polarization from pro-inflammatory M1 to anti-inflammatory M2 phenotypes in metabolic and fibrotic models. In cancer, however, it inhibits the polarization of tumor-associated M2 macrophages. Overall, KMF modulates multiple immune cell types and signaling pathways, positioning it as a promising candidate for treating autoimmune, inflammatory, and neoplastic diseases. Further translational research is warranted to explore its clinical utility and optimize delivery strategies.

## 1 Introduction

**Figure 1:**
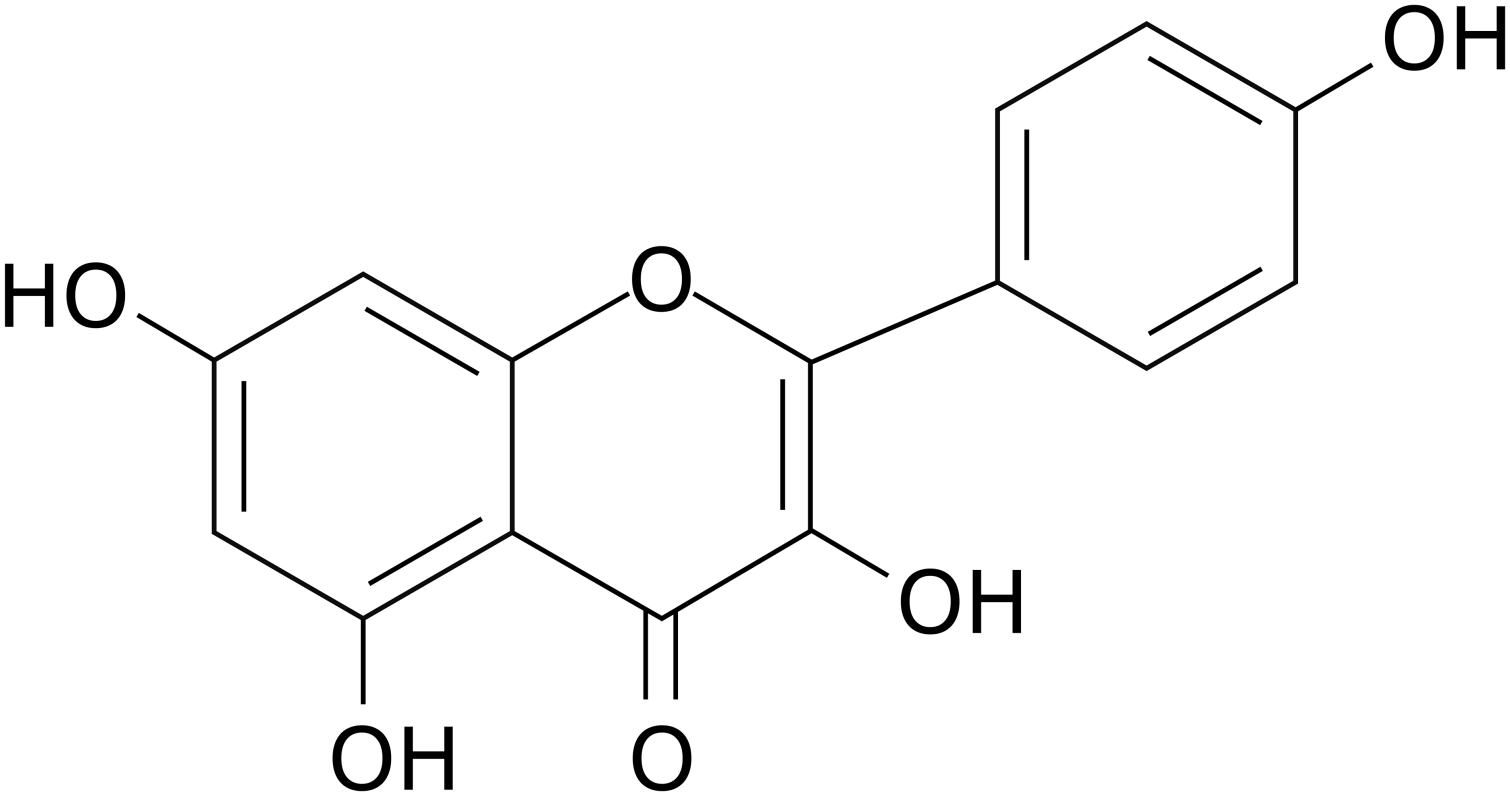
The chemical structural formula of KMF.

The immune system comprises diverse immune cells and molecules that play pivotal roles in eliminating senescent cells, dead cells, or pathogen-infected cells. Dysregulation of immune system components and/or their responses leads to multiple diseases, including inflammatory disorders, autoimmune diseases, and cancer (1–3). Modulation of aberrant immune system components and/or responses is critical for treating various diseases, such as cellular immunotherapy for cancer and molecular-targeted therapy for autoimmune diseases (4, 5). Natural products, particularly flavonoids, have emerged as promising immunomodulatory candidates due to their broad capacity to regulate multiple immune cells and signaling pathways with minimal toxicity (6, 7). Kaempferol (KMF), one of the most widely studied flavonoids, has a molecular formula of C_15_H_10_O_6_ (**Figure 1**) and a relative molecular weight of 286.24 (8). KMF is named in honor of Engelbert Kaempfer, a 17^th^-century German physician, naturalist, and historian who significantly contributed to transmitting medical knowledge from Japan to the West (9). KMF was first identified in *Camellia sinensis* and exhibits numerous health-promoting effects (9). As a yellow dietary flavonoid, subsequent studies have shown its presence in various fruits, vegetables, and medicinal herbs, including apples, beans, carrots, strawberries, saffron, and *ginkgo* leaves (10–12). A detailed account of its sources and distribution has been comprehensively summarized in one previous review (9). It is sparingly soluble in water but soluble in dimethyl sulfoxide, hot ethanol, ether, and alkali (8). KMF is absorbed in the small intestine via its lipophilicity, primarily through passive absorption, facilitated diffusion, or active transport (10, 13). Biologically, KMF and its glycosylated derivatives exhibit cardioprotective (14), neuroprotective (15), anti-inflammatory (16), antidiabetic (17), antioxidant (18), antimicrobial (9), and anti-cancer activities (19). Over the past decade, research has expanded understanding of KMF’s broad-spectrum effects on diverse immune cells, including suppression of activation, subset polarization, cytokine secretion, and infiltration via regulation of distinct molecular targets or signaling pathways (20–24). These effects suggest that KMF is a potential immunomodulator for inflammation, autoimmunity, and cancer. Regarding effective concentrations, existing studies show it exerts immunomodulatory effects, with most *in vitro* studies demonstrating efficacy at more than 1 μM despite some controversies (20, 22, 25–28). In inflammation and autoimmunity, KMF alleviates aberrant immune responses and inflammatory mediators, while it boosts anti-tumor immunity in cancer (20, 29–31). Although previous reviews have thoroughly documented KMF’s anti-tumor (19, 32), neuroprotective (33), metabolic regulatory (34), cardiovascular protective (35), and anti-infective properties (36), a comprehensive review of its modulatory effects across heterogeneous immune cell populations remains notably underexplored in current literature. To date, one review has addressed the effects of KMF on T cell subsets (37), and two reviews have included its effects on mast cells (MCs) (38, 39). However, these discussions are presented as part of broader coverage of multiple natural compounds rather than focusing exclusively on KMF. Moreover, no review has discussed the effects of KMF on other immune cell populations. This review therefore synthesizes the current understanding of KMF’s immunomodulatory effects and mechanisms across diverse immune cell types based on available literature (**Supplementary Table 1**; **Table 1**), and discusses its therapeutic potential in inflammatory and immune-related disorders.

**Table 1:** The influence of Kaempferol on different immune cells in various animal models.

Inducers	Models	Kaempferol	Influence on immune cells	Outcomes
Mite extract plus DNCB (20)	Atopic dermatitis	15 or 50 mg/kg five days a weeks for four weeks by gavage	·Mitigate the activity of effector T cells ·Ameliorate T cell activation	Improve manifestation of atopic dermatitis
Allo-antigens (29)	GVHD	20, 50, or 150 μg/body on days 0−4, 6, and 8 post transplant by ip	·Suppress cytokine secrection by T cells ·Inhibit the expansion of cytotoxic T cells	Improve clinical and pathological injuries
Allo-antigens (94)	Corneal transplantation	50 mg/kg starting 3 days before the operation and continuing till the date of sampling or till the end of observation	·Suppresse M1 macrophage polarization	Alleviate corneal rejection
Collagen (43)	Rheumatoid arthritis	200 mg/kg/day starting on day 35 for a duration of four weeks by gavage	·Decrease CD4/CD8 ratio ·Reduce CD4^+^ Tem and increase naïve/Tregs ·Promote Th2 cell differentiation	Improve arthritis symptoms
Collagen (45)	Rheumatoid arthritis	25 or 50 mg/kg from days 22 to 56 after vaccination by gavage	·Restore Th17 and Treg balance	Improve arthritis symptoms
Monosodium urate (16)	Gouty arthritis	50, 100, or 200 mg/kg for one week by ip	·Restore Th17 and Treg balance	Alleviate gouty arthritis
Imiquimod (22)	Psoriasis	9.01, 27.03, or 81.09 mg/kg for one week by gavage	·Reduce DC infiltration	Alleviate psoriasis-like skin lesions
Imiquimod (46)	Psoriasis	50 or 100 mg/kg for one week by gavage	·Restore Th17 and Treg balance	Alleviate psoriasis-like skin lesions
OVA (57)	Vaccination	50 or 100 mg/kg along with inducers by ip	·Promote the recruitment of DCs ·Promote Th1 and Th2 immune response	Adjuvant activity
OVA (72)	Allergic asthma	10 or 20 mg/kg one hour before OVA challenge by gavage	·Inhibit eosinophil recruitment ·Impaire eosinophil-epithelial interactions	Ameliorate airway inflammation
OVA (74)	Allergic asthma	20 mg/kg for three weeks by gavage	·Suppress eosinophil differentiation	Ameliorate airway inflammation
OVA (75)	Allergic rhinitis	0.2, 2, or 20 mg/kg before OVA challenge for 10 days by gavage	·Inhibit recruitment of mast cells ·Inhibit inflammation of mast cells	Ameliorate airway inflammation
High-fat diet (69)	Steatohepatitis	20 mg/kg for 12 weeks by gavage	·Inhibit neutrophil inflammation	Ameliorate non-alcoholic fatty liver
High-fat diet (58)	Intestinal inflammation	0.1% diet supplementation for 16 weeks	·Reduce DC infiltration ·Reduce macrophage/neutrophil infiltration	Improve intestinal barrier integrity and inhibit gut inflammation
Aspergillus fumigatus (27)	Fungal keratitis	1 mL (60 μg/mL) at 24 hours post-infection by ip, and 5 μL subconjunctival injections from 1 day post-infection until sacrifice	·Reduce neutrophil infiltration ·Inhibit neutrophil inflammation	Ameliorate the severity of keratitis and depress corneal fungal load
4T1 cells (23)	Tumor-bearing mice	40, 80, and 160 mg/kg for four weeks by gavage	·Inhibit NET formation ·Disrupt pro-tumorigenic effect of neutrophils	Suppress tumor growth and metastasis
Lewis lung carcinoma (30)	Tumor-bearing mice	50 mg/kg for three weeks by gavage	·Promote activation of NK cells ·Increase cytotoxic NK cells	Inhibit the growth of tumor cells
Ethanol (66)	Gastric ulcers	40, 80, or 160 mg/kg 1 h before induction of ulcers	·Reduce neutrophil infiltration ·Inhibit neutrophil inflammation	Protect the mucosa from lesions
LPS (85)	Depression-like models	25 and 50 mg/kg for two weeks by gavage	·Suppress mast cell activation	Reversed depression-like behaviors
P2 peptide (91)	EAE	50 and 100 mg/kg day 11 post-immunization to disease peak by gavage	·Reduce macrophage infiltration	Alleviate sciatic nerve symptoms and pathological injury
Insulin resistance (92)	Chronic inflammation	50 mg/kg for six weeks by gavage	·Reduce M1 macrophage polarization ·Increase M2 macrophage polarization	Reduce body weight, fat mass, and adipocyte size
CCl_4_ (24)	Liver fibrosis	50 or 100 mg/kg for six weeks by ip	·Suppresse M1 macrophage polarization	Decrease liver pathologic changes

DNCB, 2,4-Dinitrochlorobenzene; ip, intraperitoneal injection; GVHD, graft-versus-host disease; Tem, effector memory T cells; Tregs, regulatory T cells; NK cells, natural killer cells; DCs, dendritic cells; NET, neutrophil extracellular traps; OVA, ovalbumin; LPS, lipopolysaccharide; CCl_4_, Carbon tetrachloride; EAE, experimental autoimmune neuritis.

## 2 Effects of KMF on T cells

T cells are central orchestrators of adaptive immunity, with their dysregulation linked to a series of diseases, including autoimmune disorders, inflammatory conditions, and cancers (40, 41). Numerous studies have already shown that KMF is a promising modulatory agent for T cells due to its diverse biological activities, including affecting T cell activation, subset balance, and signaling pathways (20, 29, 42).

### 2.1 Regulation of T cell activation and survival

**Figure 2:**
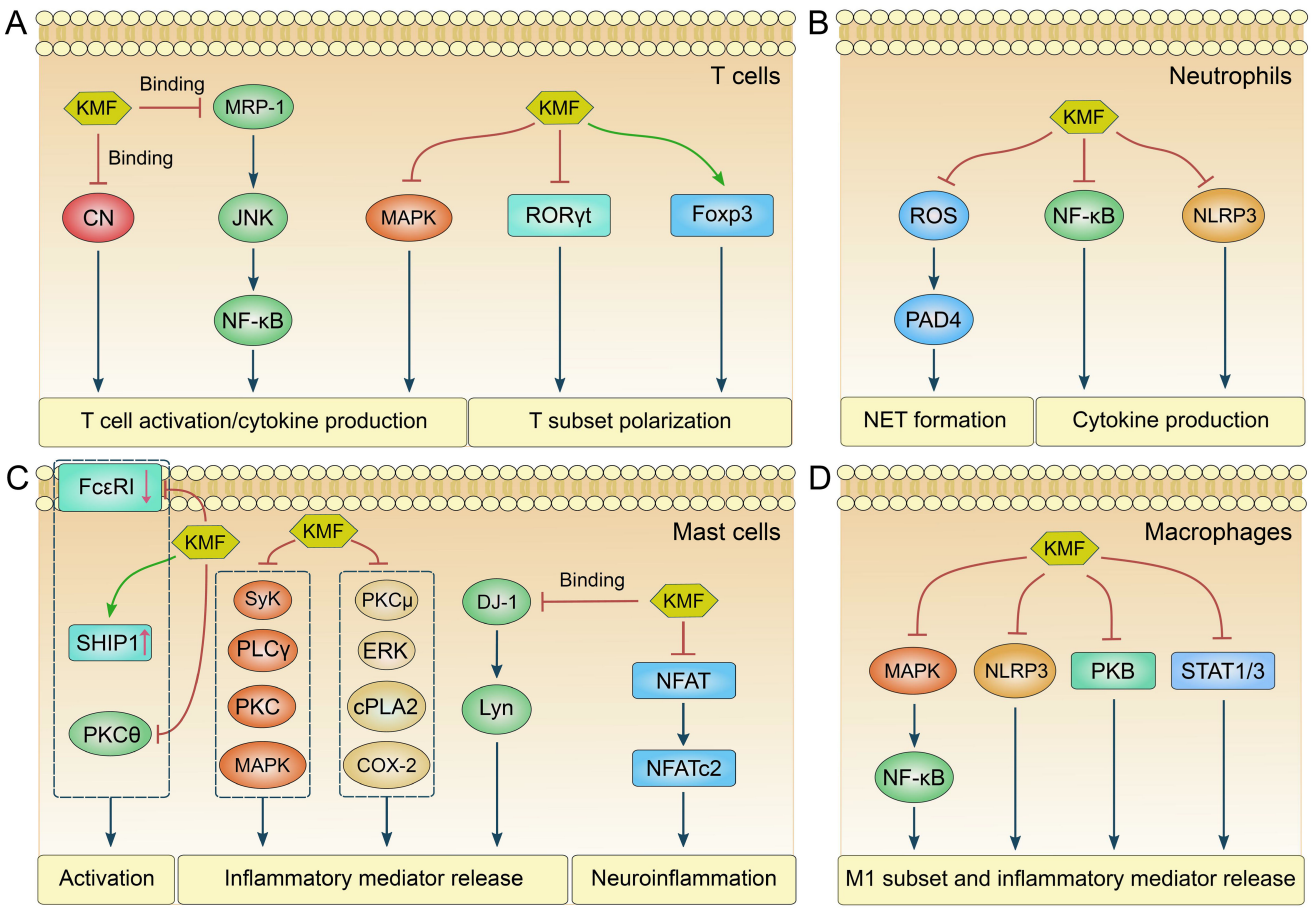
The molecular pathways regulated by KMF in different immune cells. **(A)** The major molecular pathways modulated by KMF in T cells are as follows: KMF inhibits T cell activation and cytokine production by binding to calcineurin (CN) and multidrug resistance-associated protein 1 (MRP-1), or by inactivating mitogen-activated protein kinases (MAPK). It also regulates T cell subset differentiation by suppressing retinoic acid-related orphan receptor γt (RORγt) while upregulating forkhead box p3 (Foxp3) expression. **(B)** The major molecular pathways modulated by KMF in neutrophils are as follows: KMF suppresses the formation of neutrophil extracellular traps (NET) by inhibiting the reactive oxygen species (ROS)/peptidylarginine deiminase 4 (PAD4) signaling axis. It also represses cytokine production through the inhibition of nuclear factor-κB (NF-κB) and NOD-like receptor family pyrin domain-containing 3 (NLRP3) pathways. **(C)** The major molecular pathways modulated by KMF in mast cells (MCs) are as follows: KMF inhibits MC activation by downregulating immunoglobulin E receptor (FcϵRI) expression while upregulating the expression of Src homology 2 domain-containing inositol 5-phosphatase 1 (SHIP1). Furthermore, the inactivation of PKCθ is also involved in this regulatory process. KMF suppresses the release of inflammatory mediators from MCs by inhibiting multiple signaling pathways, including spleen tyrosine kinase (Syk), phospholipase Cγ (PLCγ), PKC, MAPK, extracellular signal-regulated kinase (ERK), cytosolic phospholipase A2 (cPLA2), and cyclooxygenase-2 (COX-2). Additionally, KMF can exert its function by binding to Parkinson disease protein 7 (DJ-1). Moreover, KMF alleviates neuroinflammation through the suppression of the nuclear factor of activated T cells (NFAT) pathway. **(D)** The major molecular pathways modulated by KMF in macrophages are as follows: KMF inhibits M1 subset polarization and the release of inflammatory mediators through the suppression of multiple signaling pathways, including MAPK/NF-κB, NLRP3, protein kinase B (PKB), and STAT pathways.

**Figure 3:**
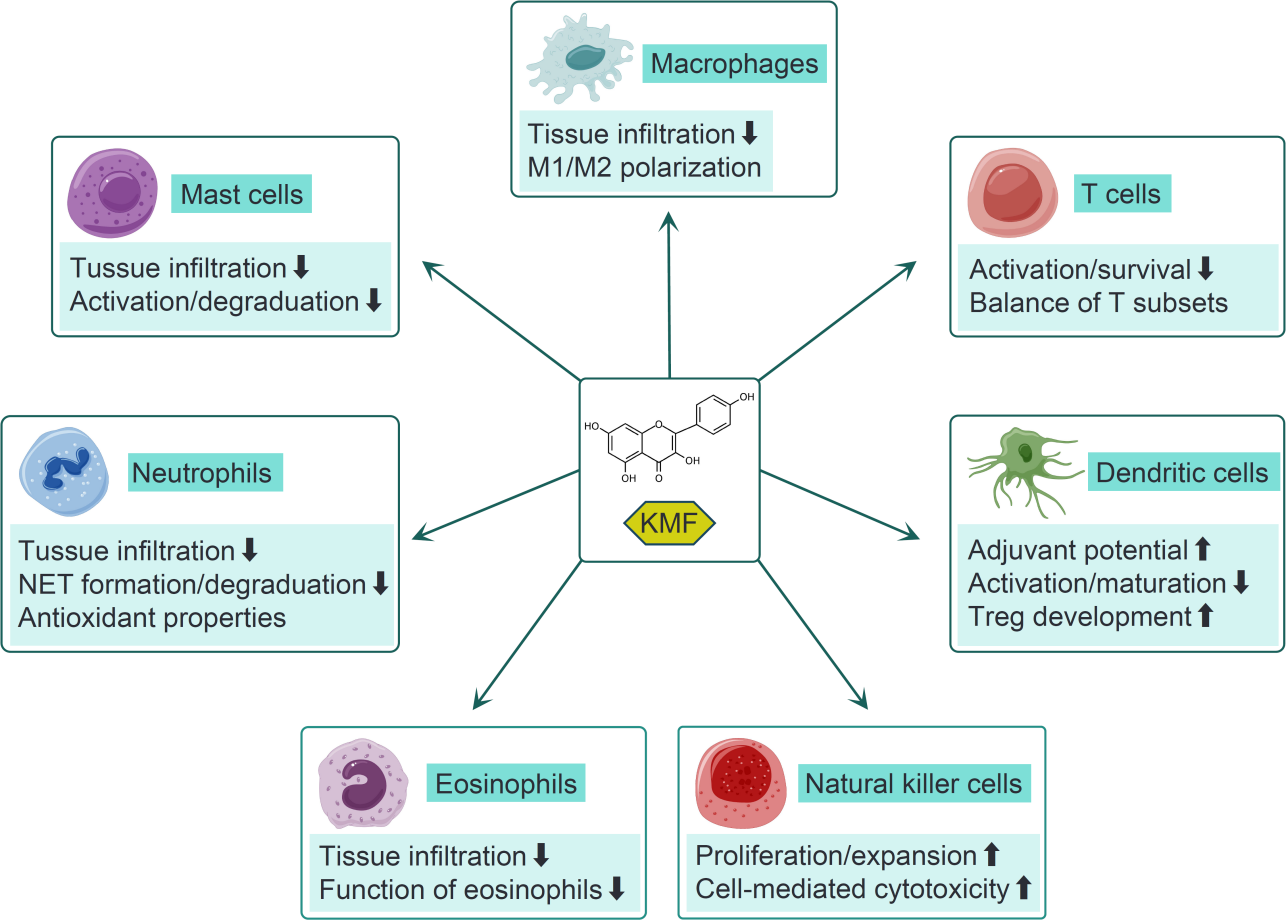
The modulatory effects of KMF on different immune cells. KMF reduces macrophage infiltration into inflamed tissues and modulates M1/M2 subset polarization across diverse disease models. It inhibits T cell activation and survival while regulating the balance among different T cell subsets. In dendritic cells (DCs), KMF enhances adjuvant potential and regulatory T cell (Treg) development, yet suppresses their activation and maturation. Additionally, KMF suppresses eosinophil recruitment and functional activity. In neutrophils, it represses tissue infiltration, the formation of neutrophil extracellular traps (NET), and degranulation processes. In mast cells (MCs), KMF inhibits their recruitment, activation, and degranulation. For natural killer (NK) cells, KMF promotes their proliferation, expansion, and cell-mediated cytotoxicity.

KMF exhibits potent inhibitory effects on T cell activation (20, 29). In a study of murine atopic dermatitis, pretreatment with KMF reduced CD69 (the earliest surface marker for activated T cells) expression and interleukin-2 (IL-2) production in activated Jurkat cells and murine CD4^+^ T cells, an effect attributed to its binding with multidrug resistance-associated protein 1 (MRP-1). This interaction suppressed c-Jun N-terminal kinase (JNK) phosphorylation and the transforming growth factor-β-activated kinase 1 (TAK1)-IκB kinase α (IKKα)/nuclear factor-κB (NF-κB) pathway, thereby improving clinical outcomes in atopic dermatitis (**Figure 2A**) (20). Similarly, KMF was proven to suppress interferon-γ (IFN-γ) and IL-2 production in T cells, and inhibit the expansion of cytotoxic CD8^+^ T cells in a graft-versus-host disease (GVHD) model. These effects were associated with reduced allospecific cytotoxic T lymphocyte (CTL) activity and mitigation of aGVHD as shown by early recovery from body weight loss, increased survival, and reduced tissue injury in the liver and large intestine (GVHD target organs) (29). In rheumatoid arthritis (RA) animal models, KMF demonstrated the efficacy in attenuating arthritis and decreased the proportion of CD4^+^ effector memory T cells (Tem) while increasing naïve and regulatory T cells (Tregs). A decreased CD4^+^/CD8^+^ T cell ratio was noted after flow cytometry analysis, indicating suppressed T cell hyperactivity. These findings suggest KMF mitigates RA pathogenesis by dampening excessive T cell activation (43). Moreover, KMF also displayed effectively modulatory effects on T cell survival and cell cycle progression. It induced mitochondria-dependent apoptosis in T cells, particularly in leukemic Jurkat T cells, leading to G2 cell cycle arrest, p53 phosphorylation, and subsequent caspase-3/8/9 activation (44). This effect was abrogated in Bcl-xL-overexpressing cells, confirming a Bcl-xL-sensitive apoptotic pathway (44). While primarily studied in cancer models, these findings suggest KMF may regulate T cell survival in immune contexts. Thus, KMF could suppress T cell activation and affect T cell fate (**Figure 3**), highlighting its multifaceted role in regulating T cell-mediated immune responses.

### 2.2 Regulation of T cell subset balance

KMF exerts significant effects in regulating T cell subset balance to promote an anti-inflammatory phenotype (**Figure 3**). In murine allogeneic hematopoietic stem cell transplantation (allo-HCT) models, KMF shifted the T helper 1 (Th1)/Th2 balance toward a Th2 phenotype, partially alleviating the progression of acute GVHD (29). Additionally, its immunomodulatory actions involved balancing Th17 and Treg subsets. In gouty arthritis, KMF restored the Th17/Treg imbalance by suppressing the expression of IL-17, tumor necrosis factor-α (TNF-α), and transforming growth factor-β1 (TGF-β1) in monosodium urate (MSU)-induced rats. This was accompanied by downregulation of retinoic acid-related orphan receptor γt (RORγt) (a Th17 transcription factor) and upregulation of forkhead box p3 (Foxp3) (a Treg marker), suggesting a shift toward anti-inflammatory immunity (16). Similarly, in a collagen-induced arthritis (CIA) mouse model, KMF showed the capacity to regulate the miR-34a/Foxp3 axis, inhibit Th17 differentiation and promote Treg expansion, thereby reducing joint inflammation and bone erosion (45). KMF reduced IL-17A^+^CD4^+^ T cell infiltration in psoriatic lesions and increased splenic/lymph node Treg frequency in imiquimod-induced psoriasis. Depletion of CD4^+^CD25^+^ Tregs abrogated these effects, confirming Treg-dependent immunosuppression (46). Therefore, these studies suggest KMF’s role in reestablishing T cell subset balance, a critical step in resolving autoimmune and inflammatory pathologies.

### 2.3 Modulation of T cell signaling pathways

KMF modulates multiple signaling pathways critical for T cell function (**Figure 2A**). It was identified as a novel calcineurin (CN) inhibitor that directly targets the CN catalytic domain to suppress *IL2* gene expression in activated Jurkat cells (42). Unlike clinically used cyclosporine A and tacrolimus, this inhibition was non-competitive and independent of immunophilins, suggesting a unique mechanism for CN-mediated T cell regulation (42). In RA, KMF inhibited the fibroblast growth factor (FGF)/FGFR3/ribosomal S6 kinase 2 (RSK2) axis, thus reducing T cell-mediated cytokine release (e.g., IL-17, IL-21, TNF-α) and fibroblast-like synoviocyte proliferation (47). Additionally, KMF affected the NF-κB and mitogen-activated protein kinases (MAPK) pathways. Park et al. reported that KMF suppressed NF-κB activation in aged rat kidneys, downregulating proinflammatory genes (*COX2*, *Nos2*) via inhibition of nuclear factor-inducing kinase (NIK)/IKK and MAPK signaling (48). Another group demonstrated that KMF enhanced Treg suppressive function by reducing proviral integration site 1 kinase (PIM1)-mediated Foxp3 phosphorylation at Ser422, increasing Foxp3 stability and expression (49). This effect correlated with improved outcomes in CIA, highlighting KMF’s role in enhancing Treg-mediated immune tolerance (49). Consequently, these studies establish KMF as a multi-target agent in T cells, offering a potential advantage over single-target drugs in preventing drug resistance.

## 3 Effects of KMF on NK cells

NK cells represent a critical component of the innate immune system, celebrated for their ability to recognize and eliminate virus-infected cells and tumor cells without prior sensitization. Their functions include direct cytotoxicity via perforin/granzyme pathways, cytokine secretion, and modulation of adaptive immunity. Dysregulated NK cell activity has been implicated in cancer progression, viral persistence, and autoimmune disorders (50–52). KMF and its derivatives have been reported to promote NK cell proliferation and cell-mediated cytotoxicity, particularly in tumor models (**Figure 3**). In a study examining phenolic compounds in *Thesium chinense* (a source of Bairui Granules), astragalin (KMF-3-O-β-d-glucopyranoside) significantly enhanced the proliferation of human umbilical cord blood-derived NK (UCB-NK) cells from 41.03% ± 0.48% to 67.22% ± 0.68% at concentrations of 8−16 μg/mL, suggesting that KMF derivatives may facilitate NK cell expansion (21). Another study investigating the antitumor effects of KMF in lung cancer xenografts demonstrated its significant activation of NK cells in mice bearing Lewis lung carcinoma (LLC). Flow cytometry analysis revealed increased proportions of cytotoxic NK cells in peripheral blood, which correlated with reduced tumor growth (30). Kaempferitrin also exhibited concentration-dependent immunostimulatory effects on NK cells, inducing an 11% increase in NK cell activity *in vitro* at the highest tested concentration (25 μM), as measured by their ability to lyse K562 target cells (53). These findings align with the traditional use of kaempferitrin-containing plants for immune enhancement, highlighting its potential to augment NK cell expansion and cytotoxicity. A notable mechanism underlying KMF-mediated NK cell activation involved gut microbiota modulation. Guan et al. reported that KMF treatment in LLC bearing mice altered the gut microbiome, increasing the abundance of beneficial bacteria such as *Lactobacillus* and *Bacteroides* species. These microbial changes correlated with enhanced NK cell and cytotoxic T cell activity, suggesting involvement of the gut-immune axis (30). KMF may promote the growth of bacteria that produce short-chain fatty acids or other metabolites, which in turn activate T and NK cell effector functions or improve their trafficking to tumor sites (30). Collectively, these findings indicate the dual role of KMF in directly enhancing NK cell functions and indirectly modulating microbial-immune interactions.

## 4 Effects of KMF on DCs

DCs are pivotal antigen-presenting cells that bridge innate and adaptive immunity, regulating immune responses through antigen presentation, cytokine secretion, and T cell priming (54). Dysregulated DC function has been implicated in inflammatory diseases, autoimmune disorders, and cancer (55, 56). KMF exhibits context-dependent effects on DC maturation and activation (**Figure 3**). In one study evaluating its adjuvant potential, KMF significantly enhanced the immune capacity of ovalbumin (OVA) and remarkably promoted the recruitment of CD11c^+^MHCII^+^ DCs in the peritoneum. Additionally, KMF treatment induced upregulation of T-bet (Th1 marker) and GATA-3 (Th2 marker) expression in splenocytes, alongside increased Th1/Th2 immune responses, indicating its role in promoting DC-mediated adaptive immune priming (57). Conversely, KMF exerted immunosuppressive effects on lipopolysaccharide (LPS)-stimulated mouse bone marrow (BM)-derived DCs, downregulating MHC class II and costimulatory molecules (CD40, CD80, CD86) while impairing DC-induced T cell activation *in vitro* and *in vivo* (25). Consistently, in an imiquimod-induced psoriasis model, KMF ameliorated skin lesions by reducing DC infiltration into the skin (22) and repressed DC recruitment in high-fat diet (HFD)-induced murine intestinal inflammation (58). Notably, conflicting evidence showed KMF had no effect on costimulatory molecule expression in LPS- and 2,4-dinitrofluorobenzene (DNFB)-stimulated BM-derived DCs (59). KMF also demonstrates significant modulation of cytokine and chemokine secretion by DCs, despite existing controversies. It inhibited proinflammatory cytokines (IL-6, IL-12p17, TNF-α) and chemokines, including monocyte chemoattractant protein-1 (MCP-1), macrophage inflammatory protein-1β (MIP-1β), and regulated on activation normal T cell expressed and secreted (RANTES), in LPS-stimulated BM-derived DCs (25). Similarly, Upadhaya et al. reported KMF could block IL-6 and IL-12 production in LPS/DNFB-stimulated DCs, although its effect on extracellular signal-regulated kinase (ERK) phosphorylation was minimal compared to other berry compounds (59). These cytokine changes likely influence T cell differentiation, with reduced IL-12 dampening Th1 responses and decreased IL-6 mitigating proinflammatory Th17 polarization. Furthermore, the water-soluble derivative astragalin-galactoside (Ast-Gal) promoted DC maturation and activation via upregulation of surface molecules (CD80, CD86, MHC class II) and increased IL-12 production to enhance Th1-mediated responses *in vitro* and *in vivo* (60). This discrepancy highlights the structural modification-dependent effects of KMF on DC cytokine profiles, where glycosylation may enhance stimulatory activities. Intriguingly, a recent study revealed KMF accelerated Treg development by inducing *Raldh2* expression in DCs via the aryl hydrocarbon receptor (AhR) and purine-rich region-binding protein 1 (PU.1)/interferon regulatory factor 4 (IRF4) pathways (26). This modulation of the DC-Treg axis suggests KMF may promote immune tolerance, a critical mechanism in autoimmune disease treatment (**Figure 3**). Thus, KMF shows context-dependent effects on DCs, modulating their maturation and activation, cytokine secretion, and T cell responses in various diseases.

## 5 Effects of KMF on neutrophils

Neutrophils, as pivotal innate immune cells, play a dual role in host defense and inflammation. They rapidly respond to infections, tissue damage, and pathological stimuli through phagocytosis, cytokine secretion, and formation of neutrophil extracellular traps (NETs) (61, 62). KMF has been shown to impede neutrophil accumulation in inflamed tissues, a critical step in alleviating pathological conditions (**Figure 3**). In a HFD-induced obesity model, KMF effectively decreased intestinal neutrophil infiltration, thereby mitigating inflammation and tissue injury (58). A KMF derivative-containing mixture attenuated neutrophil recruitment to the lungs and reduced the production of proinflammatory cytokines (IL-6, TNF-α) in LPS-induced acute lung injury models, leading to alleviation of lung inflammation (63). Similarly, in a fungal keratitis model, KMF reduced the severity of keratitis in mice, partially through inhibition of neutrophil-mediated inflammatory responses (27). The formation of NETs, a process involving the release of DNA-histone complexes, can exacerbate tissue damage in diseases such as cancer and autoimmunity (64). In a mouse breast cancer model, KMF demonstrated the ability to reduce the expression of citrullinated histone H3 (H3-cit), a specific marker of NETs (**Figure 3**), without affecting neutrophil survival (23). Mechanistically, KMF inhibited reactive oxygen species (ROS) production in BM-derived neutrophils, blocking the ROS-peptidylarginine deiminase 4 pathway, which was critical for NET formation. This highlights its potential to disrupt the pro-tumorigenic neutrophil phenotype (23). Moreover, KMF affects neutrophil degranulation (**Figure 3**), a process critical for the release of cytotoxic enzymes such as elastase and myeloperoxidase (MPO) (65). In an ethanol-induced model of gastric ulcers, KMF significantly decreased the ulcer index, increased the preventive index, completely protected the mucosa from lesions, and preserved gastric mucosal glycoprotein. The gastroprotective activity of KMF was attributed to the preservation of gastric mucous glycoprotein levels, inhibition of neutrophil infiltration and MPO activity, and regulation of pro-inflammatory cytokine levels (66). Antioxidant properties of KMF directly impact neutrophil function by reducing ROS and nitric oxide (NO) production (**Figure 3**). In short-term interactions with amoebae and hamster neutrophils, KMF treatment was associated with a reduction in ROS, NO, and MPO activities, which were speculated to be mechanisms involved in the resolution of amoebic liver abscesses (67). KMF modulates key inflammatory pathways in neutrophils, primarily through inhibition of NF-κB and NOD-like receptor family pyrin domain-containing 3 (NLRP3) inflammasome activation (**Figure 2B**). In a LPS-induced mastitis model of BALB/c mice, it strikingly reduced MPO activity, NF-κB activation, and secretion of downstream cytokines (TNF-α, IL-6, IL-1β) in mammary tissues (68). Likewise, in an HFD-induced nonalcoholic steatohepatitis (NASH) murine model, KMF attenuated hepatic injury by inhibiting neutrophil-mediated inflammation and reducing the production of TNF-α and IL-1β (69). These effects were recapitulated *in vitro*, where KMF decreased NLRP3 inflammasome components in palmitic acid-stimulated HepG2 cells, linking neutrophil-mediated inflammation to metabolic dysfunction (69). Thus, KMF inhibits neutrophil infiltration and functions in various disease models by regulating neutrophil accumulation, NET formation, antioxidant activity, and inflammatory signaling pathways.

## 6 Effects of KMF on eosinophils

Eosinophils are key effector cells in allergic and inflammatory diseases, particularly asthma and allergic rhinitis, where their excessive infiltration and activation drive airway inflammation, tissue remodeling, and symptom exacerbation (70, 71). Multiple studies have demonstrated that KMF significantly reduces eosinophil recruitment to inflamed airways, a cardinal feature of allergic inflammation (**Figure 3**) (72–74). In an OVA-induced mouse model of allergic asthma, oral KMF administration attenuated OVA challenge-elevated expression of eotaxin-1 (a key eosinophil chemokine) and eosinophil major basic protein by blocking NF-κB transactivation, thereby blunting eosinophil accumulation in airway and lung tissue (72). This effect was recapitulated in OVA-induced allergic airway inflammation in guinea pigs, where KMF reduced eosinophil counts in bronchoalveolar lavage fluid (BALF) and lung tissue, accompanied by decreased levels of the Th2 cytokines IL-5 and IL-13, critical regulators of eosinophil differentiation and survival (73, 74). Furthermore, KMF impaired eosinophil-epithelial cell interactions by suppressing eotaxin-1 and intracellular adhesion molecule-1 (ICAM-1) expression in airway epithelial cells (**Figure 3**) (72). In an OVA-induced allergic rhinitis model, KMF reduced allergic symptoms and key mediators (IgE and histamine), alongside decreased eosinophil infiltration in nasal mucosal tissue. These effects involved regulation of IL-32, thymic stromal lymphopoietin (TSLP), and caspase-1 activity (75). GRRK, a glycosylated KMF derivative, inhibited Th2 cytokine production (IL-5, IL-13) and reduced MHC class II and CD40 expression in BALF cells, attenuating eosinophil-mediated airway hyperresponsiveness (76). Astragalin, a KMF glucoside, further exemplified this activity by blocking toll-like receptor 4 (TLR4)-protein kinase Cβ2 (PKCβ2)-nicotinamide adenine dinucleotide phosphate (NADPH) oxidase signaling in airway epithelial cells, thereby inhibiting LPS-induced eotaxin-1 production and epithelial apoptosis, critical processes driving eosinophil recruitment and airway dysfunction (77). Collectively, these findings indicate that KMF disrupts eosinophil trafficking to inflamed tissues, likely by inhibiting chemokine/cytokine-driven recruitment pathways.

## 7 Effects of KMF on MCs

MCs are key effector cells in allergic and inflammatory responses, characterized by their ability to store and rapidly release histamine, cytokines, and lipid mediators upon activation, particularly via the high-affinity immunoglobulin E (IgE) receptor (FcϵRI) (78). Dysregulated MC activation contributes to conditions such as allergic asthma, atopic dermatitis, and anaphylaxis (79). IgE-mediated MC activation and degranulation are critical to multiple allergic diseases (79). KMF demonstrated dose-dependent inhibitory effects on IgE-induced degranulation and cytokine production (including IL-1β, IL-6, IL-8, IL-13, TNF-α) in various MC models (**Figure 3**), such as rat basophilic leukemia cell line 2H3 (RBL-2H3) and human mast cell-1 (HMC-1) lines, mouse BM-derived MCs (BMMCs), and human UCB-derived MCs (28, 80, 81). Mechanistically, these effects involved post-translational downregulation of the IgE receptor (FcϵRI), upregulation of Src homology 2 domain-containing inositol 5-phosphatase 1 (SHIP1), and inhibition of protein kinase Cθ (PKCθ), which collectively disrupted multiple signaling pathways driving MC activation (28, 81). In RBL-2H3 cells, KMF further reduced the secretion of β-hexosaminidase, histamine, IL-4, and TNF-α, while suppressing IgE-mediated phosphorylation of spleen tyrosine kinase (Syk), phospholipase Cγ (PLCγ), protein kinase C (PKC), and MAPK (82). *In vivo*, KMF ameliorated IgE-sensitized mouse models of passive cutaneous anaphylaxis (PCA) (82), OVA-induced allergic rhinitis (AR) (reducing IgE, histamine, and MC infiltration) (75), and OVA/IgE-induced paw swelling, hypothermia, and serum levels of histamine, TNF-α, IL-8, and MCP-1 (83). The mechanism in paw skin involved binding to Parkinson disease protein 7 (DJ-1) to inhibit full activation of Lyn kinase and downstream signaling molecules (83). KMF also suppressed β-hexosaminidase release and cyclooxygenase-2 (COX-2)-mediated production of prostaglandin D2 (PGD2) and F2α (PGF2α) in sensitized MCs by disrupting the PKCμ-ERK-cytosolic phospholipase A2 (cPLA2)-COX2 signaling axis, as well as the Syk-PLCγ pathway to inhibit airway wall thickening in antigen-exposed MCs (84). Beyond allergies, KMF exhibited effects in neuroinflammation: it reversed LPS-induced depression-like behaviors in mice by suppressing hippocampal MC activation, downregulating TNF-α via inhibiting nuclear factor of activated T cells (NFAT) transcriptional activity and NFATc2 nuclear translocation, and blocking store-operated calcium entry (SOCE) through binding to calcium release-activated calcium modulator (ORAI) (85). Additionally, KMF inhibits leukotriene B4 (LTB4) production in the mast cell line PB-3c without cytotoxicity, targeting chemical mediators critical for allergic symptoms like food allergies and hay fever (86). The major molecular pathways regulated by KMF in MCs were summarized (**Figure 2C**). Collectively, these findings highlight KMF’s multi-faceted inhibition of MC activation across allergic and neuroinflammatory contexts, via mechanisms spanning receptor regulation, signaling pathway blockade, calcium channel inhibition, and mediator suppression.

## 8 Effects of KMF on macrophages

Macrophages, as pivotal immune cells, play a central role in orchestrating both protective immune responses and pathological inflammation, with their functions tightly regulated by activation states and polarization into pro-inflammatory M1 or anti-inflammatory M2 phenotypes (87, 88). Dysregulated macrophage activity contributes to the development of various diseases, including atherosclerosis, metabolic syndrome, autoimmune disorders, and cancer (89, 90). In different disease models, KMF has been shown to alleviate inflammation and inhibit macrophage infiltration (**Figure 3**), such as obesity-associated intestinal inflammation and experimental autoimmune neuritis (58, 91). The modulation of macrophage polarization represents a key mechanism through which KMF exerts its biological effects (**Figure 3**). Chronic inflammation mediated by macrophages is crucial in insulin resistance, which could be reversed by KMF, accompanied by reduced infiltration of M1 macrophages in adipose tissue, decreased expression of pro-inflammatory markers (TNF-α, IL-1β) and increased anti-inflammatory markers (arginase-1, IL-10) (M2 phenotype) (92). This effect is mediated in part by inhibition of the NLRP3 inflammasome pathway, a central driver of sterile inflammation in obesity-related insulin resistance (92). Similarly, in carbon tetrachloride-induced liver fibrosis, KMF suppressed M1 polarization by blocking the MAPK/NF-κB axis, leading to reduced collagen deposition and alleviated hepatic inflammation (24). KMF-loaded hydrogels promoted M2 macrophage polarization and reduced matrix metalloproteinase-9 (MMP-9) expression to accelerate wound healing by balancing inflammatory and reparative responses in the diabetic wound model rats (93). Additionally, KMF could inhibit the activation of NLRP3 inflammasomes by inducing autophagy, thus inhibiting M1 macrophage polarization and ultimately alleviating corneal transplantation rejection (94). Conversely, in the context of cancer, KMF inhibited the polarization of tumor-associated macrophages (TAMs) toward the M2 phenotype, disrupting their role in promoting tumor angiogenesis and immune evasion (31). Thus, these studies suggest that KMF exhibits distinct regulatory effects on macrophage polarization in different disease models.

There are a series of investigations to explore the molecular mechanisms underlying the regulatory effects of KMF on macrophages (95–97) (**Figure 2D**). In a previous study, the effects of 36 natural flavonoids and related compounds on proinflammatory NO production were evaluated in macrophages exposed to LPS. KMF inhibited the expression of inducible nitric oxide synthase (iNOS) in a dose-dependent manner via targeting its transcription factors NF-κB and signal transducer and activator of transcription 1 (STAT1) (95). KMF attenuated IL-6-induced COX-2 expression in human monocytic THP-1 cells, suggesting its beneficial role in chronic inflammation. The mechanisms lay in that KMF deactivated and prevented nuclear localization of two major transcription factors, STAT3 and NF-κB, which are mutually responsible for COX-2 induction in response to IL-6 (96). A KMF derivative, 3-O-(2G-glucosylrutinoside)-7-O- glucoside (KGG), showed the activities to suppress inflammatory mediator release (NO, prostaglandin E2, TNF-α, IL-1β, and IL-6) in RAW macrophages via inhibiting NF-κB, MAPK, and protein kinase B pathways (97). Inhibition of NF-κB and MAPK pathways by other KMF derivatives also could be observed in RAW macrophages (98). Furthermore, KMF mitigated foam cell formation in atherosclerosis by reducing the uptake of oxidized low-density lipoprotein (ox-LDL) and enhancing cholesterol efflux through downregulation of the scavenger receptor CD36 and upregulation of cholesterol transporters, ATP-binding cassette (ABC) transporter A1 (ABCA1) and (ABCG1) (99). Mechanistically, the c-Jun-activator protein-1 (AP-1)-dependent downregulation of CD36 and the heme oxygenase-1 (HO-1)-dependent upregulation of ABCG1 and ABCA1 might mediate the beneficial effects of KMF on foam cell formation (99). The E3 ubiquitin ligase TRIM29 is highly expressed in macrophages (100), and promotes SUMOylation of protein kinase R-like endoplasmic reticulum kinase (PERK), thereby enhancing PERK expression and stability (101). Additionally, PERK has been shown to drive M2 macrophage polarization (102). While direct evidence regarding the effect of KMF on the TRIM29/PERK axis remains lacking, other flavonoids have been reported to regulate PERK expression (103, 104). Thus, KMF may modulate macrophage polarization through the TRIM29/PERK axis. Moreover, pyruvate kinase M2 (PKM2) plays a crucial role in long non-coding RNA AK083884-mediated M2 macrophage polarization (105). Inhibition of PKM2 by KMF has been observed in cancer cells (106, 107). Therefore, KMF may modulate macrophage polarization through the regulation of PKM2. Collectively, these studies indicate that KMF regulates macrophage bioactivities through different molecular targets or signaling pathways, primarily NF-κB, STAT, and MAPK.

## 9 Summary and future direction

This review systematically discusses the immunomodulatory effects of KMF on various immune cell populations and their underlying mechanisms. It exhibits significant modulatory activities by regulating the activation, subset polarization, cytokine secretion, and infiltration of different immune cells. In T cells, it inhibits activation through the JNK/NF-κB and calcineurin pathways while regulating the balance of Th1/Th2 and Th17/Treg subsets. For NK cells, KMF enhances their proliferation and cytotoxicity directly and modulates the gut microbiota-immune axis indirectly. In DCs, KMF shows context-dependent effects, either promoting adaptive immune priming or suppressing pro-inflammatory cytokine secretion. In neutrophils, eosinophils, MCs, and macrophages, KMF exerts anti-inflammatory or anti-tumor effects by inhibiting inflammatory signaling pathways, reducing cell infiltration, or regulating polarization states, with mechanisms involving NF-κB, MAPK, and NLRP3 inflammasome. Current studies on KMF exhibit several limitations, including over-reliance on animal models and *in vitro* experimental systems, which introduce potential translational gaps for extrapolation to human contexts, and insufficient exploration of its bioavailability —a critical factor for clinical applicability (108). Regarding immune-modulatory effects, both dose-dependent and structure-dependent characteristics are observed: dose dependence is evident, as seen in kaempferitrin’s concentration-dependent immunostimulatory activity on NK cells (21); structure dependence is prominent, with glycosylation and modifications altering activities like DC cytokine secretion and inflammatory mediator suppression (25, 57). Additionally, KMF’s effects vary across species (mice, rats, guinea pigs) and disease models: in autoimmune/inflammatory conditions, it predominantly exerts anti-inflammatory/immunosuppressive effects; in tumors, it enhances NK cytotoxicity and inhibits M2 polarization of tumor-associated macrophages, exerting anti-tumor immunity. Thus, critical research gaps necessitate future exploration. First, due to KMF’s poor water solubility, advanced drug delivery systems (e.g., liposomes, polymeric nanoparticles) are essential to improve solubility, stability, and targeted delivery to inflamed tissues or tumors, enhancing efficacy while minimizing toxicity. Second, despite tested dosing ranges, optimizing regimens (dose, window, duration) requires pharmacokinetic profiling and time-dependent effect studies to clarify differences between prophylactic and therapeutic administrations. Mechanistically, while associations with immune cell changes are documented, deeper validation of cell-specific mechanisms, via CRISPR-Cas9, proteomics, or single-cell RNA sequencing, is needed to clarify causal pathways. Additionally, systematic structure-activity relationship studies of derivatives are lacking; future work should synthesize modified analogs to improve solubility/tissue specificity and explore synergies with existing immunotherapies via high-throughput screening. Finally, translating preclinical findings requires humanized models (humanized mice, patient-derived organoids/xenografts) and multi-omics to identify biomarkers, alongside early-phase trials to evaluate safety/efficacy. Integrating nanotechnology, systems biology, and translational approaches positions KMF as a promising low-toxicity, high-efficacy immunomodulator for immune-related diseases.

## Glossary

KMF: kaempferol
MRP-1: multidrug resistance-associated protein 1
JNK: c-Jun N-terminal kinase
IKKα: IκB kinase α
NF-κB: nuclear factor-κB
IFN-γ: interferon-γ
IL-2: interleukin-2
GVHD: graft-versus-host disease
CTL: cytotoxic T lymphocyte
RA: rheumatoid arthritis
Tem: effector memory T cells
Tregs: regulatory T cells
Th1: T helper 1
TNF-α: tumor necrosis factor-α
TGF-β1: transforming growth factor-β1
RORγt: retinoic acid-related orphan receptor γt
Foxp3: forkhead box p3
CIA: collagen-induced arthritis
CN: calcineurin
FGF: fibroblast growth factor
RSK2: ribosomal S6 kinase 2
MAPK: mitogen-activated protein kinases
NIK: nuclear factor-inducing kinase
PIM1: proviral integration site 1 kinase
NK cells: natural killer cells
UCB: umbilical cord blood
LLC: Lewis lung carcinoma
DCs: dendritic cells
LPS: lipopolysaccharide
BM: bone marrow
DNFB: 2,4-dinitrofluorobenzene
MCP-1: monocyte chemoattractant protein-1
MIP-1β: macrophage inflammatory protein-1β
RANTES: regulated upon activation, normal T cell expressed and secreted
ERK: extracellular signal-regulated kinase
Ast-Gal: astragalin-galactoside
AhR: aryl hydrocarbon receptor
PU.1: purine-rich region-binding protein 1
IRF4: interferon regulatory factor 4
NETs: neutrophil extracellular traps
H3-cit: citrullinated histone H3
ROS: reactive oxygen species
MPO: myeloperoxidase
NO: nitric oxide
NLRP3: NOD-like receptor family pyrin domain-containing 3
NASH: nonalcoholic steatohepatitis
ICAM-1: intracellular adhesion molecule-1
OVA: ovalbumin
TLR4: toll-like receptor 4
PKC: protein kinase C
NADPH: nicotinamide adenine dinucleotide phosphate
MCs: mast cells
FcϵRI: immunoglobulin E receptor
RBL-2H3: rat basophilic leukemia cell line 2H3
HMC-1: human mast cell-1
BMMCs: BM-derived mast cells
SHIP1: Src homology 2 domain-containing inositol 5-phosphatase 1
Syk: spleen tyrosine kinase
PLCγ: phospholipase Cγ
PCA: passive cutaneous anaphylaxis
NFAT: nuclear factor of activated T cells
SOCE: store-operated calcium entry
LTB4: leukotriene B4
MMP-9: metalloproteinase-9.
