# Supplementary material for: Kaempferol as a multifaceted immunomodulator: implications for inflammation, autoimmunity, and cancer
Source: Front Immunol. 2025 Aug 21;16:1671519. doi: 10.3389/fimmu.2025.1671519 (PMC12408509; doi:10.3389/fimmu.2025.1671519)
Supplement: Supplementary file 1 [file DataSheet1.pdf]

Supplemental Table 1 Common and unique signaling pathway molecules modulated by KMF in different immune cells

**Different signaling pathway molecules**	
Common signaling pathway molecules	
Nuclear factor-κB (NF-κB)	
Mitogen-activated protein kinases (MAPK)	
NOD-like receptor family pyrin domain-containing 3 (NLRP3)	
Unique signaling pathway molecules	
T cells	Calcineurin (CN); Multidrug resistance-associated protein 1 (MRP-1); c-Jun N-terminal kinase (JNK); Retinoic acid-related orphan receptor γt (RORγt); Forkhead box p3 (Foxp3)
Dendritic cells	Purine-rich region-binding protein 1 (PU.1)/Interferon regulatory factor 4 (IRF4); T-bet; GATA-3
Neutrophils	Reactive oxygen species (ROS)/Peptidylarginine deiminase 4 (PAD4)
Eosinophils	Toll-like receptor 4 (TLR4)-Protein kinase Cβ2 (PKCβ2)-nicotinamide adenine dinucleotide phosphate (NADPH)
Mast cells	Immunoglobulin E receptor (FcεRI); Src homology 2 domain-containing inositol 5-phosphatase 1 (SHIP1); PKCθ; Spleen tyrosine kinase (Syk); Phospholipase Cγ (PLCγ); PKC; Extracellular signal-regulated kinase (ERK); Cytosolic phospholipase A2 (cPLA2); Cyclooxygenase-2 (COX-2)
Macrophages	Protein kinase B (PKB); Signal transducer and activator of transcription (STAT)
